# Detention in Juvenile Correctional Facilities Is Associated with Higher Platelet Monoamine Oxidase B Activity in Males

**DOI:** 10.3390/biom10111555

**Published:** 2020-11-15

**Authors:** Josip Podobnik, Matea Nikolac Perkovic, Gordana Nedic Erjavec, Katarina Dodig Curkovic, Mario Curkovic, Vlatka Kovac, Dubravka Svob Strac, Melita Cusek, Marco Bortolato, Nela Pivac

**Affiliations:** 1 Department of Psychiatry, Psychiatric Hospital for Children and Youth Zagreb, Kukuljeviceva 11, 10000 Zagreb, Croatia; josippodobnik@yahoo.com; 2 Division of Molecular Medicine, Ruder Boskovic Institute, Bijenicka cesta 54, 10000 Zagreb, Croatia; mnikolac@irb.hr (M.N.P.); gnedic@irb.hr (G.N.E.); dsvob@irb.hr (D.S.S.); 3 Department for Child and Adolescent Psychiatry, Clinical Hospital Center Osijek, J. Huttlera 4, 31000 Osijek, Croatia; dodig-curkovic.katarina@kbo.hr (K.D.C.); vlatka.kovac@yahoo.com (V.K.); 4 Family Medicine, Osijek Health Center, Park Kralja Petra Krešimira IV. 6, 31000 Osijek, Croatia; mario.curkovic@dzo.hr; 5 Juvenile Correctional Facility Ivanec, Pahinsko 6, 42240 Ivanec, Croatia; mcusek@socskrb.hr; 6 Department of Pharmacology & Toxicology, College of Pharmacy, Salt Lake City, 30 South 2000 East, UT 84112, USA; marco.bortolato@utah.edu

**Keywords:** juvenile correctional facility, delinquent behavior, platelet MAO-B activity, *MAOB* rs1799836 polymorphism, conduct disorder

## Abstract

Juvenile delinquency is related to several biological factors, yet very few vulnerability biomarkers have been identified. Previous data suggest that the enzyme monoamine oxidase B (MAO-B) influences several personality traits linked to the propensity to engage in delinquent behavior. Building on this evidence, we assessed whether conduct disorder (CD), juvenile delinquency adjudications, or detention in a correctional facility were associated with either platelet MAO-B activity or the *MAOB* rs1799836 polymorphism. The study enrolled 289 medication-free male youths, including 182 individuals detained in a correctional facility (with or without a diagnosis of CD). Of the remaining 107 participants, 26 subjects had a diagnosis of CD, and 81 were mentally healthy controls. Platelet MAO-B activity was determined by spectrophotofluorometry, while *MAOB* rs1799836 was genotyped using qPCR. Platelet MAO-B activity, corrected for age and smoking, was significantly higher in juvenile detainees (*p* < 0.001), irrespective of CD diagnosis. *MAOB* rs1799836 was not associated with platelet MAO-B activity or with detention in a correctional facility, CD diagnosis, or delinquent behavior. These data suggest that detention in a juvenile correctional facility increases platelet MAO-B activity in male adolescents. Future studies are needed to determine the mechanisms and functional significance of MAO-B peripheral elevation in juvenile male detainees.

## 1. Introduction

Conduct disorder (CD) is a persistent pattern of antisocial and disruptive behaviors in children and adolescents, characterized by harmful behavior towards animals and people, disregard for the rights and properties of others, and violation of societal norms [1,2,3]. In line with male preponderance of aggression and antisocial behavior, the prevalence rate of CD is higher in males than females [3,4]. Given the robust association of CD with juvenile delinquency, about 38% of juvenile male detainees meet diagnostic criteria for this disorder [5].

Recent evidence has shown that the ontogeny of CD and delinquent behavior is robustly influenced by biological factors [6,7], yet the molecular underpinnings of this condition remain poorly understood. Previous research has shown that both internalizing and externalizing psychopathologies in male juvenile delinquents were associated with low platelet activity of monoamine oxidase type B (MAO-B) [8], one of the two enzymes that catalyze the oxidative deamination of monoaminergic neurotransmitters [9]. Consistently, reduced platelet MAO-B activity was found to be associated with delinquent and criminal behaviors [10,11], poor impulse control, extraversion, as well as predisposition for risky, impulsive, sensation-seeking, and novelty-seeking behaviors [10,12,13]. In keeping with these results, genetic deficiency of MAO-B in mice was found to be associated with behavioral disinhibition [14].

Preliminary evidence supports that platelet MAO-B activity may be a heritable factor [15]. One of the best-characterized *MAOB* polymorphisms is rs1799836, consisting of an A to G substitution, and located in intron 13 of this gene, 36 bp upstream from the boundary with exon 14 [16]. This polymorphism has been associated with different risk for CD-related psychopathological states. For example, Netter and colleagues [17] reported that, in alcohol-dependent patients, the G genotype was associated with higher aggression scores than A- allele carriers, even though this finding was not confirmed by a study performed on a much larger cohort [18].

Building on these premises, this study was aimed at evaluating whether platelet MAO-B activity and *MAOB* rs1799836 polymorphism were associated with CD diagnosis, delinquency (defined as a conviction for criminal offenses), and detention in a correctional facility in a cohort of male youths.

## 2. Materials and Methods

### 2.1. Participants

This study included 289 young male drug-naive subjects, subdivided as follows: 182 subjects detained in the Juvenile Correctional Facility Ivanec, Zagreb County, Croatia (age: 16.7 ± 0.18 years old), with (*N* = 117) and without (*N* = 65) diagnosis of CD; 26 outpatients (i.e., out of detention) with CD (age: 15.0 ± 0.41 years old); and 81 mentally healthy controls (13.7 ± 0.24 years old). The last two cohorts were recruited during their regular medical examination at the Family Medicine Unit in Osijek, Croatia, or the Department of Child and Adolescent Psychiatry in the Clinical Hospital Centre, Osijek.

Among the juvenile detainees, 86 had no history of convictions, while the other 96 (hereafter referred to as “subjects with court convictions”) had records of delinquency adjudications. To evaluate different aggressive/dissociative behaviors, Hare Psychopathy Checklist: Youth Version (PCL-YV), Overt Aggression Scale-Modified (OAS-M), and Child Behavior Checklist (CBCL) were used [19,20,21].

Diagnoses of CD were confirmed by a team of child psychiatrists, and a psychologist, according to the Structured Clinical Interview for DSM-IV criteria (SCID-IV) [22]. Smoking status was determined by a questionnaire; subjects were classified as smokers (*N* = 124) or nonsmokers (*N* = 165). All participants were medication-free. The following exclusion criteria were used: use of psychoactive compounds or alcohol; diagnosis of autism spectrum disorder, intellectual disability or ADHD, and a treatment history of cognitive behavioral therapy or electroconvulsive therapy. Control youth did not have any psychiatric diagnosis. All participants gave voluntary informed consent to be a part of the study. The study was approved by the Ethics Committees in each institution, and was fully compliant with the ethical standards laid down in the 1975 Declaration of Helsinki.

### 2.2. Determination of Platelet MAO-B Activity

Whole blood samples were collected in 8.5 mL yellow-top Vacutainer tubes (Becton, Dickinson and Company, Franklin Lakes, NJ, USA) with 1.5 mL of acid citrate dextrose anticoagulant. Sampling was done in the morning around 8 a.m., after overnight fasting. Platelets were obtained from whole blood by a series of centrifugation steps and broken by sonication. Platelet MAO-B activity was determined in all participants as previously described [23] with a slightly modified method described by Krajl [24] and using kynuramine (Sigma-Aldrich, St. Louis, MS, USA) as a substrate. Spectrophotofluorometric determination of MAO-B was performed on a Varian Cary Eclipse fluorescence spectrophotometer (Varian, Palo Alto, CA, USA), with excitation wavelength of 310 nm and emission range of 380 nm. Platelet protein levels were measured by the method of Lowry [25]. Platelet MAO-B activity was calculated using the standard curve of 4-hydroxyquinoline (4-OHQ; Sigma-Aldrich, St. Louis, MS, USA) and expressed in nmol of 4-OHQ formed per hour, per mg of total platelet proteins.

### 2.3. Genotyping of MAOB rs1799836 Polymorphism

Extraction of genomic DNA was carried out with the salting-out method [26]. *MAOB* intron 13 A/G polymorphism (rs1799836) was determined with real-time PCR according to the procedures described by Applied Biosystems. Genotyping was done with an ABI Prism 7300 Real-time PCR System apparatus (Applied Biosystems, Foster City, CA, USA), using primers and probes from Applied Biosystems (Foster City, CA, USA) as TaqMan^®^ SNP Genotyping Assay (C_8878790_10). Graphical presentation of real-time PCR data is shown as supplementary material (Figure S1). Genotyping was done by a researcher blinded to all clinical data. Around 10% of the samples were re-genotyped for quality control.

### 2.4. Statistical Analysis

Results were expressed as numbers and percentages or median and range (minimum and maximum) and evaluated with Sigma Stat 3.5 (Jandel Scientific Corp., San Jose, CA, USA). Normality of distribution was assessed with the Kolmogorov–Smirnov test. Multiple linear regression analysis was used to assess the influence of CD diagnosis, age, smoking, and *MAOB* rs1799836 on platelet MAO-B activity. Comparison of platelet MAO-B activity and age between different groups of subjects was made using Kruskal–Wallis ANOVA by ranks or Mann–Whitney U test, since normality of the data failed. Allele frequencies were compared using χ2-test and Yates correction for continuity. As the study included only male subjects, we could not determine Hardy–Weinberg equilibrium for *MAOB* rs1799836, since *MAOB* is located on the X chromosome. All tests were two-tailed, and α was set at 0.025 (as we tested the significance of two variables, namely platelet MAO-B and *MAOB* rs1799836).

G*Power 3 Software [27] was used to determine a priori sample size. For ANOVA (with α = 0.025; power (1−β) = 0.800; medium effect size (ω = 0.25) and 4 groups), total desired sample size was 180; for genetic analyses and χ2-test (with α = 0.025; power (1−β) = 0.800 and medium effect size (ω = 0.30); with df = 1, total desired sample size was 106; and with df = 2, total desired sample size was 128; for Mann–Whitney test (with α = 0.025; power (1−β) = 0.800; medium effect size (ω = 0.50), total desired sample size was 128; and for multiple regression analysis (with α = 0.025; power (1−β) = 0.800; medium effect size (ω = 0.15); number of predictors = 3), total desired sample size was 91. Therefore, the study included an adequate sample size, and the statistical power was appropriate to detect significant differences in the studied groups.

## 3. Results

Given that age and smoking have a potent effect in increasing and reducing MAO-B activity, respectively [10,28,29], we controlled for these factors as in our previous analyses [18,23,30]. Juvenile detainees with or without CD were significantly older (F = 94.30; df = 3; *p* < 0.0001; *p* < 0.001; Kruskal–Wallis ANOVA) than patients with CD or healthy controls. Furthermore, the number of smokers was higher than that of nonsmokers (χ2 = 139.21; df = 3; *p* < 0.0001). To rule out potential confounding effects of age and smoking on platelet MAO-B activity, multiple linear regression analysis was performed, with platelet MAO-B activity as the dependent variable and age and smoking as independent variables. This analysis showed no significant effects of age (*p* = 0.343) or smoking (*p* = 0.619) on platelet MAO-B activity (F(2,286) = 0.453; *p* = 0.636; Radj2 = −0.004). Thus, in further analyses, groups were not subdivided into smokers and nonsmokers or according to the age of participants.

**Figure 1:**
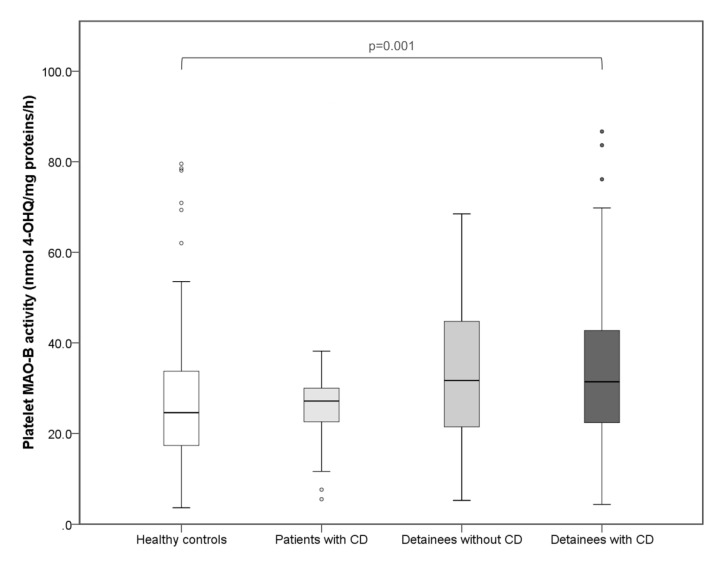
Platelet MAO-B activity in subjects detained in a juvenile correctional facility, with (*N* = 117) and without (*N* = 65) diagnosis of CD, 26 outpatients with CD, and 81 mentally healthy controls. Results are presented as a box and whisker plot. The median is represented by the line in the box, while the interquartile range (IQR) box represents the middle quartiles (the 75th minus the 25th percentile). The whiskers on either side of the IQR box represent the lowest and highest quartiles of the data. The ends of the whiskers represent the maximum and minimum of the data, while the individual dots beyond the whiskers represent outliers in the data set.

Platelet MAO-B activity was not related to a diagnosis of CD, since it did not differ significantly between youths from a correctional facility with or without CD (*p* = 0.755), or between healthy subjects and patients with CD (*p* = 0.988). Conversely, our analyses showed that this index was associated with correctional facility detention. Indeed, platelet MAO-B activity was significantly higher (H = 14.088; df = 3; *p* = 0.003; Kruskal–Wallis ANOVA) in detainees with or without CD compared to healthy controls and patients with CD (Figure 1). Dunn’s test revealed that detainees with (*p* = 0.001) and without CD (*p* = 0.011) had significantly higher platelet MAO-B activity than healthy controls. Platelet MAO-B was also significantly increased in all 182 detainees compared to 81 healthy controls (U = 9,276.50; *p* = 0.001; Mann–Whitney test). A statistical trend for higher platelet MAO-B activity (*p* = 0.030, not fully significant due to the *p* correction) was also evidenced in the comparison of detainees vs. outpatients with CD.

**Figure 2:**
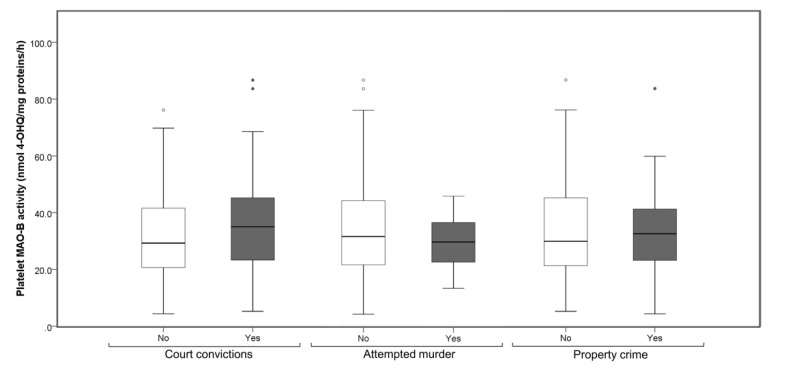
Platelet MAO-B activity in subjects detained in a juvenile correctional facility, subdivided into youths with or without court convictions, detainees with or without a record of attempted murder or detainees who committed or who did not commit property crime. Results are presented as a box and whisker plot. The median is represented by the line in the box, while the interquartile range (IQR) box represents the middle quartiles (the 75th minus the 25th percentile). The whiskers on either side of the IQR box represent the lowest and highest quartiles of the data. The ends of the whiskers represent the maximum and minimum of the data, while the individual dots beyond the whiskers represent outliers in the data set.

To further analyze the association between delinquency record and platelet MAO-B activity, detainees were subdivided based on their court adjudication. Platelet MAO-B activity did not differ significantly (Figure 2) between youths with or without court convictions (U = 4795.0; *p* = 0.060; Mann–Whitney test). Further, platelet MAO-B activity did not differ (Figure 2) between detainees with or without a record of attempted murder (U = 1619.0; *p* = 0.375, Mann–Whitney test) or between detainees who committed or who did not commit property crime (U = 3845.5; *p* = 0.838; Mann–Whitney test).

To evaluate why higher platelet MAO-B activity was associated only with juvenile detention, we studied the potential association between platelet MAO-B activity and dissociative/aggressive/delinquent behavior (determined by PCL-YV, OAS-M and CBCL) in all detainees (Table 1) using multiple linear regression. This analysis revealed that platelet MAO-B activity, corrected for smoking and group affiliation (healthy subjects vs. patients with CD vs. youths from a correctional facility with or without CD), was significantly affected (F(2,180) = 6.95; *p* = 0.001; Radj2 = 0.09) only by the scores in OAS-M item verbal aggression (*p* = 0.016), but not by other symptoms/scores, as determined by the PCL-YV, other OAS-M items, and CBCL (Table 1). These results collectively suggest that living in a correctional facility was the main factor associated with increased platelet MAO-B activity.

To evaluate whether the presence of the G or A allele of the *MAOB* rs1799836 polymorphism affects platelet MAO-B activity, we performed a multiple linear regression analysis, with platelet MAO-B activity as the dependent variable and group affiliation (healthy subjects vs. patients with CD vs. youths from a correctional facility with or without CD) and *MAOB* rs1799836 as independent variables. In agreement with previous results, this analysis revealed no significant effect of *MAOB* rs1799836 polymorphism (*p* = 0.746) and a significant effect of group affiliation (*p* = 0.004) on platelet MAO-B activity (F(2,284) = 4.31; *p* = 0.014; Radj2 = −0.023).

The frequency (χ^2^ test) of the A and G alleles of *MAOB* rs1799836 did not differ significantly (*p* = 0.810) between healthy controls, youths with CD from a correctional facility, juvenile detainees without CD, and patients with CD (Table 2), or between detainees with or without court convictions (*p* = 0.862), detainees who did or who did not attempt to commit murder (*p* = 0.167) or detainees who committed or who did not commit property crime (*p* = 0.734; Table 3).

To evaluate the possible association between *MAOB* rs1799836 alleles with delinquent behavior, multiple linear regression, corrected for smoking and group affiliation (healthy subjects vs. patients with CD vs. youths from a correctional facility with or without CD), was used, with *MAOB* rs1799836 as the independent variable and the scores on PCL-YV, OAS-M and CBCL as dependent variables in all detainees (Table 4). The results of multiple linear regression with *MAOB* rs1799836 and PCL-YV, OAS-M, and CBCL scores in detainees revealed that *MAOB* rs1799836 was not significantly (Table 4) associated with the presence of aggressive/dissociative/delinquent symptoms or behaviors assessed by these scales. These results collectively suggested that *MAOB* rs1799836 was not significantly associated with juvenile detention, diagnosis of CD, and/or delinquent behavior in male youths.

## 4. Discussion

This study examined the association of platelet MAO-B activity and *MAOB* rs1799836 polymorphism with CD, delinquent behavior, and juvenile detention in male youths, as compared with CD outpatients and healthy controls. Our main findings showed that while platelet MAO-B activity was not associated with either CD or a positive history of delinquency, it was associated with detention in a correctional facility, irrespective of a diagnosis of CD. This finding persisted even after platelet MAO-B activity was controlled for the effect of smoking and age. Finally, no associations were found between these indices and *MAOB* rs1799836.

The lack of significant associations between CD and platelet MAO-B activity confirms and expands previous evidence documenting the lack of associations between this index with symptoms of disruptive behavior symptoms in boys [31] or with measures of childhood behavioral disorders in forensic psychiatric patients with a history of recidivistic violent crimes [32].

We detected increased platelet MAO-B activity in association with juvenile detention. On the surface, these results may appear at odds with previous findings on the lower platelet MAO-B activity in imprisoned individuals who committed violent crimes [33,34,35], former juvenile delinquents who developed persistent criminality in adulthood [36], violent male prisoners detained for homicide, assault, or robbery [37], and male prisoners with a history of heavy criminality and with personality disorders (paranoid, schizotypal and schizoid, antisocial, borderline, histrionic, and narcissistic) [35]. Nonetheless, our data substantiated that the elevation in MAO-B activity was observed in detained subjects irrespective of their delinquency record, raising the possibility that higher MAO-B levels are a consequence of, rather than a risk factor for, detention in a correctional facility. In line with this interpretation, we found no association of MAO-B activity with delinquent behavior, as revealed by their delinquency adjudication record, or specific crimes, such as attempted murder or property violation. Furthermore, MAO-B activity was not associated with any index of different facets of delinquent behavior and psychopathy, with the only exception of verbal aggression evaluated by the OAS-M. This finding is also in accord with previous results showing a lack of association between platelet MAO-B and violent acts (homicide or not), history of suicide attempt, and history of heroin use [37], or indices of psychosocial adversity or crime-related factors, such as scores on the Life History of Aggression Scale and the Psychopathy Checklist [32], or with or without criminal recidivism [11]. It should be stated, however, that other reports documented a correlation of platelet MAO-B activity with criminal behavior in adolescents exposed to early life stress; this correlation disappeared in adolescents living in more favorable conditions, suggesting that this interaction is affected by an improved environment [38,39]. On the other hand, self-reported engagement in risk-taking behaviors, corresponding to a subtype of impulsivity, was associated with higher platelet MAO-B activity in adult risky drivers [40]. In the same vein, platelet MAO-B activity was found to be negatively correlated with expressed interpersonal violence in male suicide attempters [41], even though this index was not affected by aggression against self in this study. Several factors may account for these divergences, including the developmental stage of participants (namely, juvenile vs. adult offenders), the seriousness of the committed criminal acts, the specific clinical scales, or the size of the cohorts included in the studies. There are scarce data in the literature regarding the role of MAO-B and its activity in the development of antisocial behavior and behavioral disorders in female subjects. Malmberg and colleagues [31] reported an association between lower platelet MAO-B activity and symptoms of oppositional defiant disorder. However, previously mentioned studies detected no significant correlation between MAO-B activity and exposure to and/or expression of interpersonal violence during childhood in female suicide attempters [41].

The idea that detention in a correctional facility may result in biological changes, such as MAO-B activity elevation, is supported by several studies showing that this biochemical parameter is broadly associated with several psychopathological conditions. Custody in juvenile halls has a marked impact on poor health outcomes and a greater risk of psychopathology [42]. Youth in the correctional justice system with a history of mental problems had higher levels of mental health symptoms and higher scores on the symptoms listed in Brief Symptom Inventory (somatization, obsessive-compulsive disorder, interpersonal sensitivity, depression, anxiety, paranoid ideation, and psychoticism) than adult prisoners or the general population [43].

MAO-B oxidizes primarily trace amines, such as β-phenylethylamine, but also dopamine and tyramine [44]. However, in tissues and cells where its isoenzyme MAO-A is absent, such as platelets, MAO-B takes its role, including the degradation of serotonin and norepinephrine [45]. While MAO-B is most abundant in the brain (where it serves 80% of total MAO activity), platelets are one of its major peripheral sources [45]. Platelets are used as easily obtainable noninvasive peripheral models of central serotonergic neurons [46], since they share some similar processes and contain some identical components [47,48]. In view of these arguments, it has been proposed that platelet MAO-B activity can be considered as a proxy of central serotonergic functions [10,39]. At the same time, however, the relationship between MAO-B activity and alterations in monoamine contents is likely bidirectional, depending on the origin and nature of these aberrances. For example, while a reduction in MAO-B levels driven by genetic alterations is expected to produce elevations in several monoamine levels (depending on the specific local contribution of this enzyme to monoamine metabolism) [14], increased plasma concentrations of monoamine neurotransmitters may result in compensatory upregulation of MAO-B, particularly in cells that do not display MAO-A, such as platelets. This perspective suggests that both increased and decreased platelet MAO-B activity may be associated with similar deviations of blood monoamine levels, as well as the corresponding psychopathological trait. As mentioned above, rich evidence has shown that multiple neuropsychiatric conditions and behavioral phenotypes have been associated with both elevations and reductions of platelet MAO-B in comparison with nonaffected controls [8,12,23,30,45,49,50,51,52,53,54,55,56,57,58,59,60]. Thus, given the high prevalence of psychopathology among detainees [61], it is likely that our finding of elevated MAO-B activity may signify a higher risk of mental disorders in this population.

*MAOB* rs1799836 polymorphism was not significantly associated with CD, living in a juvenile correctional facility, court convictions, attempted murder or committed property crime, or the PCL-YV, OAS-M, and CBCL scores. In line with this finding, *MAOB* rs1799836 was not related to autism in children [62,63], severe agitation in patients with schizophrenia, CD, PTSD [23,30], anger-related traits and aggression in suicidal participants and controls [64], aggression determined using the Brown–Goodwin questionnaire [65], alcohol dependence [18], positive symptoms in schizophrenia [66], or negative emotionality in healthy subjects [67]. In contrast to our data, a moderate association of the G allele with higher scores on the Spontaneous Aggression subscale from the Freiburg Aggression Scale in alcohol-dependent patients [17], and the association with the development of schizophrenia in Han Chinese patients [68], but not in Turkish patients [69], was reported. *MAOB* rs1799836 might not be directly associated with CD or with delinquent behavior, but in a haplotype analysis with other polymorphisms, such as *MAOA* rs6323T [68] or *MAOB* rs1799836, rs10521432, rs6651806, and rs590551 [67], it might show significant association with altered behaviors. Differences in ethnicity, heterogeneity in the diagnoses and sample characteristics [70], gender, age, smaller sample size of cases/controls leading to lower power analysis might explain the divergent results. As recently summarized [6], in a complicated entity such as CD, some association studies proposed a number of suggestive signals, but with great inconsistencies across the studies. In addition, CD is associated with complex interaction of genetic and environmental factors [6]. To dissect this complex disease, we also evaluated the association of PCL-YV, OAS-M, and CBCL scores with *MAOB* rs1799836. However, we could not confirm the significant association of *MAOB* rs1799836 with complex psychiatric and behavioral outcomes in children and adolescents, such as CD (present study), ADHD or autism [62], alcohol dependence [18] or PTSD [29,30] in adults. Therefore, we are reporting negative genetic results, but corrected for all possible confounders. Our present and previous results and the results from other studies suggest no relationship between *MAOB* rs1799836 polymorphism and CD, living in a juvenile correctional facility, court convictions, or criminal/delinquent behavior. This is in line with results from the GWAS study that detected no evidence of significant association between any of the MAO genes and CD [71].

In agreement with all our and other previous data [17,18,23,28,29,30,72], platelet MAO-B activity was not affected by the presence of the A or G allele of the *MAOB* rs1799836 polymorphism, and temper previous evidence in vitro [73,74] or in a small number of subjects [75] that supported a possible role of this polymorphism on MAO-B activity.

Strengths of this study include the inclusion of medication-free ethnically homogeneous youth, determination of one biochemical and one genetic marker, male gender, detailed clinical evaluation of the diagnosis, symptoms, and behaviors by child psychiatrists and a psychologist, the use of PCL-YV, OAS-M, and CBCL to measure delinquent behavior and psychopathy, statistical power set to 0.80, the needed sample size and detailed statistical analysis. Conversely, key limitations of this study are that our genetic analyses were limited to only one polymorphism in the *MAOB* gene and that we were not able to include female subjects. Although the total sample size was adequate (*N* = 289), division into specific groups with unequal sample sizes (117 subjects detained in the juvenile correctional facility with CD and 65 without the diagnosis of CD, 26 outpatients with CD, and 81 mentally healthy controls) should be considered as another possible limitation of the study, especially while exploring a possible association of CD or delinquent behavior with *MAOB* rs1799836 polymorphism.

## 5. Conclusions

In conclusion, our study revealed that platelet MAO-B activity was significantly higher in detained youths, but was not affected by smoking, age, and presence of CD. It was associated with verbal aggression and with living in a correctional facility. *MAOB* rs1799836 polymorphism was not associated with CD-related phenotypes. Our data should be used for larger meta-analyses and confirmed or rejected in independent studies. If these data are confirmed, future prospective studies are warranted to elucidate whether the observed changes in platelet MAO-B activity reflect a biological consequence of, or a risk factor for juvenile detention, and elucidate the underlying mechanisms.

## Figures and Tables

**Table 1:** Multiple linear regression with platelet MAO-B activity and number of scores of aggressive/dissociative/delinquent behaviors evaluated using Hare Psychopathy Checklist: Youth Version (PCL-YV), Overt Aggression Scale-Modified (OAS-M), and Child Behavior Checklist (CBCL) in detainees.

Scales for Evaluating Dissociative/Aggressive/Delinquent Behavior	MAO-B Activity			Multiple Linear Regression Model		
	Coefficient	t	*p*	Corrected R^2^	F	*p*
PCL-YV: total	0.001	0.01	0.994	0.060	4.81	0.003
Factor 1	0.596	1.22	0.222	0.067	5.36	0.001
Factor 2	0.224	0.49	0.625	0.061	4.91	0.003
Factor 3	−0.121	−0.27	0.791	0.060	4.85	0.003
Factor 4	−0.939	−1.65	0.101	0.074	5.80	0.001
(F1+F2)/(F3+F4)	0.816	0.56	0.573	0.061	4.94	0.003
OAS-M: total	0.100	1.39	0.168	0.070	5.52	0.001
Verbal aggression	0.669	2.43	0.016	0.090	6.95	<0.001
Aggression against property	0.051	0.24	0.807	0.060	4.85	0.003
Physical aggression against property or objects	0.201	1.27	0.206	0.068	5.40	0.001
Physical aggression against self	0.115	0.42	0.672	0.061	4.91	0.003
CBCL: total	0.001	0.02	0.982	0.060	4.82	0.003
Delinquency (D)	0.182	0.71	0.478	0.062	5.01	0.002
Aggression (A)	−0.027	−0.19	0.849	0.060	4.84	0.003
A/D	−0.01	−0.02	0.986	0.063	4.96	0.003

**Table 2:** *MAOB* rs1799836 allele frequency in male medication-naive youths who were subdivided into healthy controls, young patients with conduct disorder (CD), and youths from a juvenile correctional facility without CD and with CD.

Subjects		*MAOB* rs1799836	
		Allele A N (%)	Allele G N (%)
Healthy controls		46 (56.8)	35 (43.2)
Youth with CD		14 (56.0)	11 (44.0)
Youth from the juvenile correctional facility	Without CD	37 (56.9)	28 (43.1)
	With CD	59 (50.9)	57 (49.1)
Test statistics		Χ^2^ = 0.96; df = 3; *p* = 0.810	

**Table 3:** *MAOB* rs1799836 allele frequency in male medication-naive youths who were subdivided into youths with or without court convictions, detainees with or without a record of attempted murder, and detainees who committed or who did not commit property crime.

Youth from the Juvenile Correctional Facility		*MAOB* rs1799836		χ^2^ test (df = 1)	
		Allele A N (%)	Allele G N (%)	χ^2^	*p*
Court convictions	No	44 (51.8)	41 (48.2)	0.03	0.862
	Yes	52 (54.2)	44 (45.8)		
Attempted murder	No	81 (50.9)	78 (49.1)	1.67	0.167
	Yes	15 (68.2)	7 (31.8)		
Property crime	No	61 (51.7)	57 (48.3)	0.12	0.734
	Yes	35 (55.6)	28 (44.4)		

**Table 4:** Multiple linear regression with *MAOB* rs1799836 and number of scores of aggressive/dissociative/delinquent behaviors evaluated using Hare Psychopathy Checklist: Youth Version (PCL-YV), Overt Aggression Scale-Modified (OAS-M), and Child Behavior Checklist (CBCL) in detainees.

Scales for Evaluating Dissociative/Aggressive/Delinquent Behavior	*MAOB* rs1799836			Multiple Linear Regression Model		
	Coefficient	t	*p*	Corrected R^2^	F	*p*
PCL-YV: total	−0.674	−0.60	0.550	0.433	69.61	<0.001
Factor 1	−0.059	−0.17	0.864	0.331	45.42	<0.001
Factor 2	0.035	0.09	0.925	0.244	30.11	<0.001
Factor 3	−0.550	−1.49	0.137	0.314	42.14	<0.001
Factor 4	−0.111	−0.38	0.707	0.471	81.06	<0.001
(F1+F2)/(F3+F4)	0.147	1.25	0.214	0.001	1.07	0.346
OAS-M: total	0.280	0.47	0.641	0.191	22.23	<0.001
Verbal aggression	0.739	0.91	0.363	0.137	15.30	<0.001
Aggression against Property	−1.102	−1.04	0.299	0.153	17.23	<0.001
Physical aggression against property or objects	−0.244	−0.34	0.698	0.072	8.01	<0.001
CBCL: total	−2.762	−0.92	0.358	0.328	44.83	<0.001
Delinquency (D)	−0.301	−0.45	0.654	0.417	65.36	<0.001
Aggression (A)	−0.297	−0.25	0.805	0.393	59.15	<0.001
A/D	0.251	0.77	0.444	−0.008	0.30	0.743

## Supplementary Materials

The following are available online at https://www.mdpi.com/2218-273X/10/11/1555/s1, Figure S1. Graphical representation of the results obtained after *MAOB* rs1799836 genotyping with real-time PCR.
