# Supplementary material for: Detention in Juvenile Correctional Facilities Is Associated with Higher Platelet Monoamine Oxidase B Activity in Males
Source: Biomolecules. 2020 Nov 15;10(11):1555. doi: 10.3390/biom10111555 (PMC7697475; doi:10.3390/biom10111555)
Supplement: Supplementary file 1 [file biomolecules-10-01555-s001.pdf]

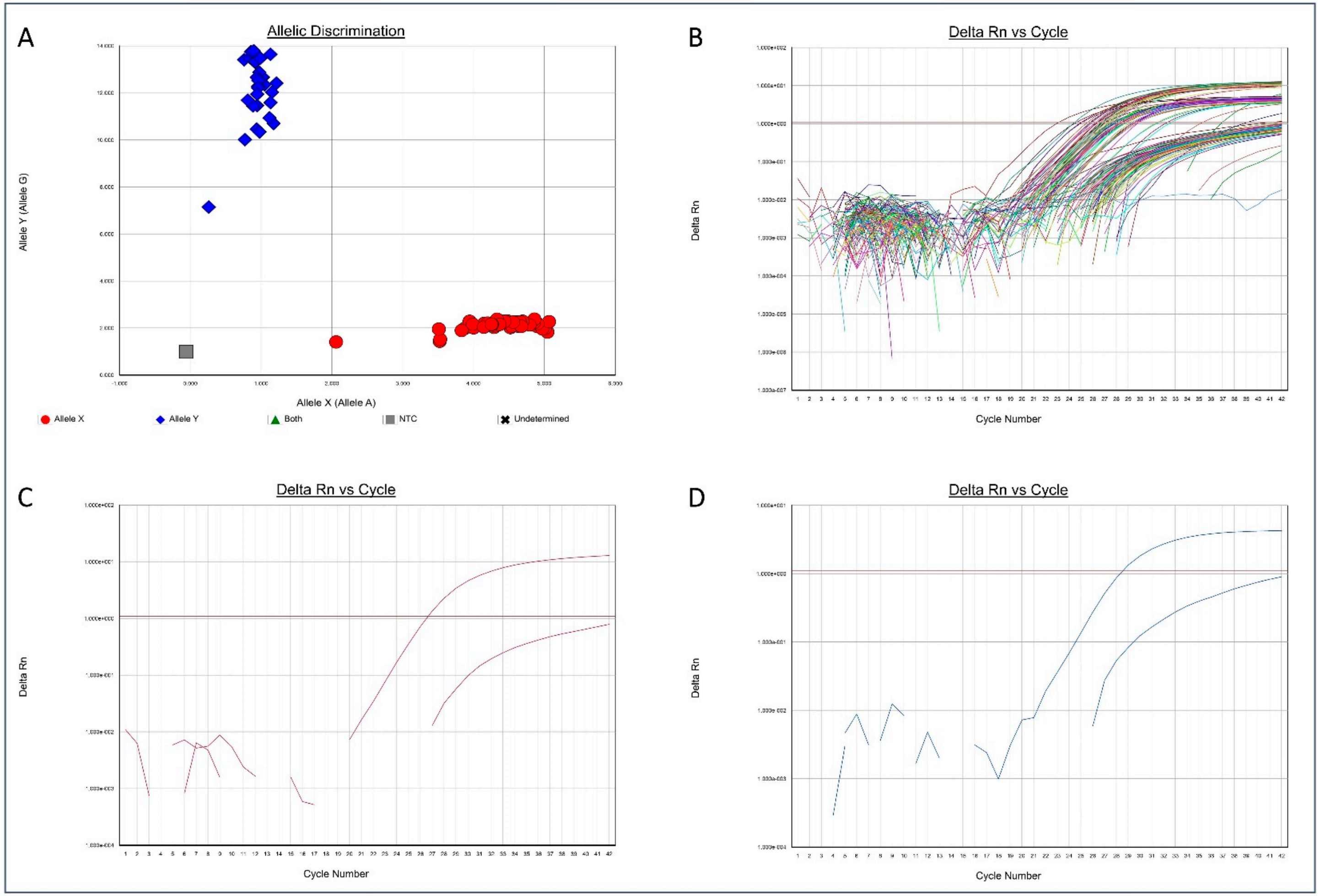

Figure S1. Graphical representation of the results obtained after *MAOB* rs1799836 genotyping with real time PCR. (**A**) Allelic discrimination method: a scatter plot representing the fluorescence of one allele plotted against the other. (**B**) Amplification plot: delta Rn value plotted against PCR cycle number for all the samples. (**C**) Amplification plot: delta Rn value plotted against PCR cycle number for A allele carrier. (**D**) Amplification plot: delta Rn value plotted against PCR cycle number for G allele carrier. Rn is the fluorescence of the reporter dye divided by the fluorescence of a passive reference dye, and delta Rn is Rn minus the baseline.
